# Long noncoding RNA ADIRF antisense RNA 1 upregulates insulin receptor substrate 1 to decrease the aggressiveness of osteosarcoma by sponging microRNA-761

**DOI:** 10.1080/21655979.2021.2019872

**Published:** 2022-01-14

**Authors:** Lingling Xu, Yinling Tan, Fengxia Xu, Yong Zhang

**Affiliations:** a Department of Oncology, Weifang Yidu Central Hospital, Weifang, Shandong China; b Department of Orthopedics, Weifang Yidu Central Hospital, Weifang, Shandong China; c Department of Orthopedics, The Fifth People’s Hospital of Jinan, Shandong China

**Keywords:** Microrna-761, adirf-as1, ceRNA theory, irs1

## Abstract

An increasing number of studies have supported the critical regulatory actions of long noncoding RNAs (lncRNAs) in osteosarcoma (OS). However, the detailed roles of adipogenesis regulatory factor-antisense RNA 1 (ADIRF-AS1) in OS have not been comprehensively described. Hence, we first detected ADIRF-AS1 expression in OS and evaluated its clinical significance. Functional experiments were then performed to determine the modulatory role of ADIRF-AS1 in OS progression. ADIRF-AS1 was found to be overexpressed in OS, and the overall survival of patients with OS who had high ADIRF-AS1 levels was shorter than that of those with low levels. ADIRF-AS1 knockdown led to restricted proliferation, migration, and invasiveness of OS cells and increased apoptosis. Additionally, ADIRF-AS1 downregulation impeded tumor growth *in vivo*. Mechanistically, ADIRF-AS1 acted as a competitive endogenous RNA for microRNA-761 (miR-761) that siphoned miR-761 away from its target, namely insulin receptor substrate 1 (IRS1), leading to IRS1 overexpression. Rescue experiments showed that low levels of miR-761 or restoration of IRS1 could neutralize the effects of ADIRF-AS1 ablation in OS cells. In summary, ADIRF-AS1 exacerbates the oncogenicity of the OS cells by targeting the miR-761/IRS1 axis. Our findings may aid in the advancement of lncRNA-directed therapeutics for OS.

## Introduction

Osteosarcoma (OS), which is derived from primitive mesenchymal cells, is the most common type of bone tumor and comprises over 20% of all primary bone malignancies [1]. It ranks second in cancer-associated mortality in children and adolescents [2]. Currently, surgery in parallel with auxiliary radiotherapy, chemotherapy, and gene therapy is the main available treatment regimen for OS [3]. Due to advancements in diagnostic and therapeutic strategies, the five-year disease-free survival rate of patients with OS has increased to 70%[4]. However, the clinical efficacy of treatments in patients who experience local/distant metastasis or recurrence remains unsatisfactory [5]. Although recent progress in understanding the molecular biology of tumors has provided novel clues into OS pathogenesis [6,7], the detailed mechanisms of OS oncogenesis and progression are far from clear. Therefore, additional research in this area may aid the development of promising OS management methods.

Long noncoding RNAs (lncRNAs) are a family of RNA transcripts over 200 nucleotides without protein-coding capacity [8]. Formerly, lncRNAs were regarded as junk sequences [9], but in recent decades, as genome sequencing technologies have improved, they have been found to participate in gene expression control and have important functions in almost all aspects of cell biology [10,11]. LncRNA dysregulation significantly correlates with the occurrence and progression of various human cancers [12], including OS [13]. Accumulating evidence has confirmed that lncRNAs play carcinogenic or anti-oncogenic roles and exhibit remarkable regulatory functions related to the aggressive biological behavior of OS [14–16].

microRNAs (miRNAs) are a group of approximately 17–24-nucleotide-long noncoding, single-stranded RNA transcripts [17] that downregulate gene expression by cleaving mRNAs or decreasing translation [18]. Many studies have reported the expression and functions of miRNAs in OS, and highlighted their considerable role in regulating its malignancy [19–21]. Multiple mechanisms used by lncRNAs have been recognized, among which the competitive endogenous RNA (ceRNA) theory has drawn much research interest [22]. LncRNAs can lower the levels of certain miRNAs by means of competitive direct binding, thus siphoning miRNAs away from their target genes, leading to mRNA overexpression [23]. Thus, lncRNAs and miRNAs contribute to osteosarcomagenesis and progression, and advancements in our knowledge of these molecules may help exploit clinically attractive targets for OS therapy.

Adipogenesis regulatory factor (ADIRF,), also known as C10orf116 or APM2, was underexpressed in gastric cancer and manifested a significant relationship with higher pathological stage, higher clinical stage, lymph node metastasis, and poorer distant relapse-free survival [24]. Furthermore, APM2 was certified as a new regulator of cisplatin resistance in many human cancer types, regardless of p53 or mismatch repairMMR status [25].

Through The Cancer Genome Atlascancer genome atlas (TCGA) database, a variety of lncRNAs were found to be differentially expressed in OS, including ADIRF antisense RNA 1 (ADIRF-AS1). ADIRF-AS1, located at chr10:86,965,287–86,971,311, has been certified as a metabolism-related lncRNA signature predicting the prognosis of patients with colorectal cancer [26]. Although many lncRNAs have been investigated, to date, no reports have been published on the roles of ADIRF-AS1 in OS. Therefore, we first detected ADIRF-AS1 expression in OS and evaluated its clinical significance. Then, functional experiments were performed to observe the modulatory role of ADIRF-AS1 in OS progression. We hypothesized that ADIRF-AS1 exacerbates the oncogenicity of OS cells by targeting the miR-761/IRS1 axis. Our observations may promote the development of ADIRF-AS1-directed OS management strategiesmanagements.

## Materials and methods

### Patients and tissue samples

The Ethics Committee of Weifang Yidu Central Hospital approved our study. All patients provided written informed consent. OS tissues and adjacent normal tissues were collected from 57 patients in the aforementioned hospital. No patients were treated with chemotherapy or radiotherapy prior to surgical resection.

### Cell cultures

The normal human osteoblast cell line hFOB 1.19 (ATCC; Manassas, VA, USA) was cultured in a 1:1 mixture of Ham’s F12/DMEM medium containing 0.3 mg/ml G418 and 10% fetal bovine serum (FBS) (Gibco; Thermo Fisher Scientific, Inc.). hFOB 1.19 cells were maintained at 33.5°C in a humidified incubator with 5% CO_2_.

Three human OS cell lines (MG-63, U-2OS and Saos-2) were purchased from the National Collection of Authenticated Cell Cultures (Shanghai, China). Saos-2 and U-2OS cells were grown in 10% FBS-supplemented McCoy’s 5A medium (Gibco). Minimum Essential Medium (Gibco) containing 10% FBS was used to culture MG-63 and HOS (ATCC) cells. A 1% penicillin-streptomycin mixture was used for culturing all cells. All OS cells were cultured at 37°C in a humidified atmosphere with 5% CO_2_.

### Cell transfection

Two different small interfering RNAs (siRNAs) against ADIRF-AS1 expression (si-ADIRF-AS1) were designed for RNA inference, with negative control (NC) siRNA (si-NC) used as a control. miR-761 oligonucleotides, including a miR-761 mimic, an NC mimic, a miR-761 inhibitor, and an NC inhibitor, were purchased from GenePharma Company (Shanghai, China). The insulin receptor substrate 1 (IRS1) overexpression vector pcDNA3.1-IRS1 was synthesized by GenScript Biotech Corp. (Nanjing, China). Lipofectamine® 2000 (Invitrogen) was used for cell transfection.

### Quantitative real-time polymerase chain reaction (qRT-PCR)

For the detection of ADIRF-AS1 and IRS1, total RNA was extracted with TRIzol® reagents (Invitrogen) and reverse-transcribed into complementary DNA utilizing a PrimeScript Reagent Kit with gDNA Eraser (Takara). Next, PCR amplification was executed with a PrimeScript™ RT Master Mix (Takara). Glyceraldehydeglyceraldehyde-3-phosphate dehydrogenase (GAPDH) served as a normalization control.

To quantify miR-761 expression, small RNA was isolated by means of RNAiso for Small RNA (Takara Biotechnology Co., Ltd). Reverse transcription was performed with a miScript Reverse Transcription Kit, and PCR amplification was completed with a miScript SYBR Green PCR Kit (both from Qiagen GmbH, Hilden, Germany). miR-761 level was normalized to that of U6. All data were analyzed using the 2^−ΔΔCq^ method.

### Cell counting kit-8 (CCK-8) assay

The CCK-8 assay was performedimplanted as previously described [27]. After 24 h of transfection, cells were collected and seeded onto 96-well plates at a density of 2000 cells per well. To assess proliferation, cells were incubated with 10 µl of CCK-8 solution (Dojindo Laboratories, Tokyo, Japan) at 37°C for 2 h. The optical density at 450 nm (OD450) was detected with a TECAN Infinite M200 multimode reader (Tecan, Mechelen, Belgium).

### Flow cytometry analysis for cell apoptosis assessment

Flow cytometrycytometric analysis was performed as previouslyimplemented as described [28]. After 48 hours, cells were digested with trypsin without ethylenediaminetetraacetic acid (EDTA) and collected for cell apoptosis assessment using an Annexin V-FITC Apoptosis Detection Kit (Beyotime; Shanghai, China). The harvested cells were washed with phosphate-buffered saline. Cells were resuspended in 195 μl Annexin V-FITC binding buffer and transfected cells were stained with 5 µl of Annexin V-FITC and 10 µl of Propidium Iodide (PI) at room temperature for 30 min away from the light. Apoptotic cells were analyzed with a flow cytometer (BD Biosciences).

### Transwell migration and invasion assays

Transwell assays were performed according to a previous study [29]. Transwell chambers (8.0 μm; BD Biosciences) were used to detect the cell migration and invasion abilities. A FBS-free culture medium was used to prepare single-cell suspensions. For the migration assay, 200 µl of cell suspension containing 5 × 10^4^ cells were seeded into the upper chambers. For the invasion assay, membranes in the upper chambers were coated with Matrigel (Corning), and the same number of cells was seeded into the upper chambers. The lower chambers were filled with 500 µl culture medium supplemented with 20% FBS. After 24 h of culture, the non-migrated and non-invaded cells were cleaned by scrubbing with a cotton swab. The migrated and invaded cells were fixed in 4% paraformaldehyde, stained with 0.5% crystal violet and photographed on an inverted microscope (Olympus).

### Xenograft experiments

Animal experiments were performed with approval from the Animal Care and Use Committee of Weifang Yidu Central Hospital. Short hairpin RNA (shRNA) against ADIRF-AS1 (sh-ADIRF-AS1) and NC shRNA (sh-NC) were inserted into a lentiviral plasmid, followed by transfection into 293 T cells. Supernatants harboring sh-ADIRF-AS1 or sh-NC lentivirus were harvested at 48 h post-transfection and used to infect U-2OS cells. Puromycin was then used to screen U-2OS cells with a stable ADIRF-AS1 knockdown. For the tumor growth study, BALB/c nude mice aged 4–6 weeks were purchased from Hunan SJA Laboratory Animal Co., Ltd. (Hunan, China). All mice were randomly classified into the sh-ADIRF-AS1 or sh-NC groups. Mice in the sh-ADIRF-AS1 group were subcutaneously injected with U-2OS cells with stably transfected sh-ADIRF-AS1. U-2OS cells overexpressing sh-NC were used as control cells in the sh-NC group. Tumor size was recorded weekly, and tumor volume was determined according to the formula: 0.5 × length × width^2^. Five weeks after treatment, all mice were euthanized, and tumor xenografts were excised, weighed, and photographed.

### Bioinformatics analysis

ENCORI (http://starbase.sysu.edu.cn/) and miRDB (http://mirdb.org/) were used to conduct a biosignal analysis to identify miRNAs downstream of ADIRF-AS1. The TargetScan website (http://www.targetscan.org), miRDB, and ENCORI were used to predict the downstream targets of miR-761.

### Subcellular fractionation

The assay was conducted as shown before [30]. OS cells in the logarithmic growth phase were harvested, and their nuclear and cytoplasmic fractions were separated with a Protein and RNA Isolation System Kit (Thermo Fisher Scientific, Inc.). RNA from the nuclear and cytoplasmic fractions was extracted and analyzed by qRT-PCR to determine the relative ADIRF-AS1 distribution in OS cells.

### RNA immunoprecipitation (RIP)

RIP was carried outrealized as previouslyprevious reported [31]. A Magna RIP RNA-Binding Protein Immunoprecipitation Kit (Millipore, Billerica, MA, USA) was used for the assay. Briefly, OS cells were scraped off the culture plates and incubated with RIP lysis buffer. Lysed cells (100 μl) were incubated with a magnetic bead-antibody complex in RIP immunoprecipitation buffer with human anti-anti-argonaute RISC catalytic component 2 (Ago2) or anti-IgG antibodies (Millipore). After overnight incubation with rotation at 4°C, the magnetic beads were collected and rinsed with wash buffer. Next, proteinase K was incubated with the immunoprecipitated complex with shaking to digest the protein. Purified immunoprecipitated RNA was assessed by qRT-PCR.

### Luciferase reporter assay

The luciferase assay was performed as in a previous study [32]. Fragments of ADIRF-AS1 harboring the predicted target site of miR-761 were amplified and cloned into the pmirGLO reporter plasmid (Promega Corporation, Madison, WI, USA), which is referred to as wild-type-ADIRF-AS1 (wt-ADIRF-AS1). The ADIRF-AS1 fragments carrying the mutant (mut) predicted target site of miR-761 were inserted into the pmirGLO reporter plasmid, which yielded mut-ADIRF-AS1. The wt-IRS1 and mut-IRS1 reporter plasmids were designed and constructed in a similar manner. OS cells were co-transfected with miR-761 mimic or NC mimic and wt or mut reporter plasmids using Lipofectamine® 2000. After 48 h, the luciferase activity was measured in accordance with the protocol of the dual-luciferase reporter analysis system (Promega Corporation).

### Western blot

As described by Feng et al [33]., cells were collected and immersed in RIPA lysis buffer (Solarbio, Beijing, China) and supplemented with phenylmethanesulfonyl fluoride. A BCA Protein Assay Kit was used to determine the protein concentration. Equal amounts of protein were subjected to 10% SDS-PAGE and then transferred to a polyacrylamide difluoride membrane. After blocking with 5% nonfat milk for 2 h and subsequent incubation with primary antibodies at 4°C overnight, membranes were probed with HRP-labeled secondary antibody (ab150077; Abcam) at room temperature for 2 h. The immunoreactive bands were detected with an enhanced chemiluminescence (ECL) system (Pierce). The following primary antibodies were used in this study: anti-IRS1 (ab40777; Abcam) and anti-GAPDH (ab181602; Abcam).

### Statistical analysis

All results were obtained from at least three independent experiments. Data were expressed as the mean ± standard deviation. The student’s t test was used for comparisons between two groups. One-way analysis of variance with Tukey’s post hoc test was employed to detect differences among multiple groups. The Kaplan–Meier method and log-rank test were used to assess the relationship between ADIRF-AS1 expression and the overall survival of patients with OS. Pearson’s correlation coefficient analysis was applied to detect gene expression correlations. P values less than 0.05 indicated statistical significance.

## Results

In the present study, we aimed to investigateexplore the expression status and clinical relevancemeaning of ADIRF-AS1 in OS. Additionally, the detailed rolesroles and underlying mechanisms of ADIRF-AS1 in OS were systematically describedelaborated.

### ADIRF-AS1 is highly expressed in OS and indicates a poor prognosis

**Figure 1.**
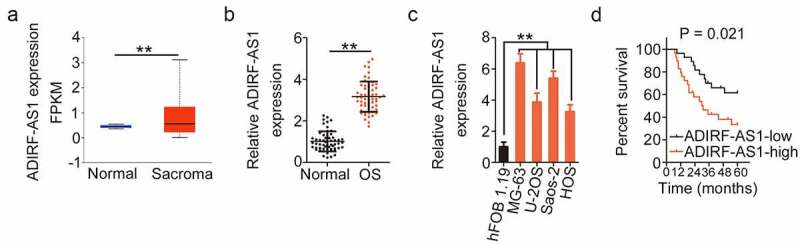
ADIRF-AS1 is upregulated in OS. (a) ADIRF-AS1 levels in sarcoma were evaluated using the TCGA database. (b) The expression level of ADIRF-AS1 in OS tissues. (c) DIRF-AS1 levels in OS cell lines. (d) Kaplan–Meier curve of overall survival evaluating the relationship between the ADIRF-AS1 level and overall survival of patients with OS. **P < 0.01.

To determine whether ADIRF-AS1 correlated with OS progression, its expression in sarcoma was first analyzed in the TCGA database. Compared with normal tissues, ADIRF-AS1 was significantly overexpressed in sarcoma (Figure 1a). As assessed by qRT-PCR, ADIRF-AS1 was highly expressed in OS tissues relative to adjacent normal tissues (Figure 1b). qRT-PCR also detected a higher ADIRF-AS1 expression in a panel of OS cell lines compared with its expression in hFOB 1.19 (Figure 1c). Then, using the median value of ADIRF-AS1 in OS tissues as a cutoff, all patients were divided into the ADIRF-AS1-low (n = 28) or ADIRF-AS1-high (n = 29) groups. The overall survival was shorter in the ADIRF-AS1-high group than it was in the ADIRF-AS1-low group (Figure 1d). Altogether, high ADIRF-AS1 expression in OS indicates poor prognosis.

### Downregulated ADIRF-AS1 suppresses the aggressive behavior of OS cells

**Figure 2.**
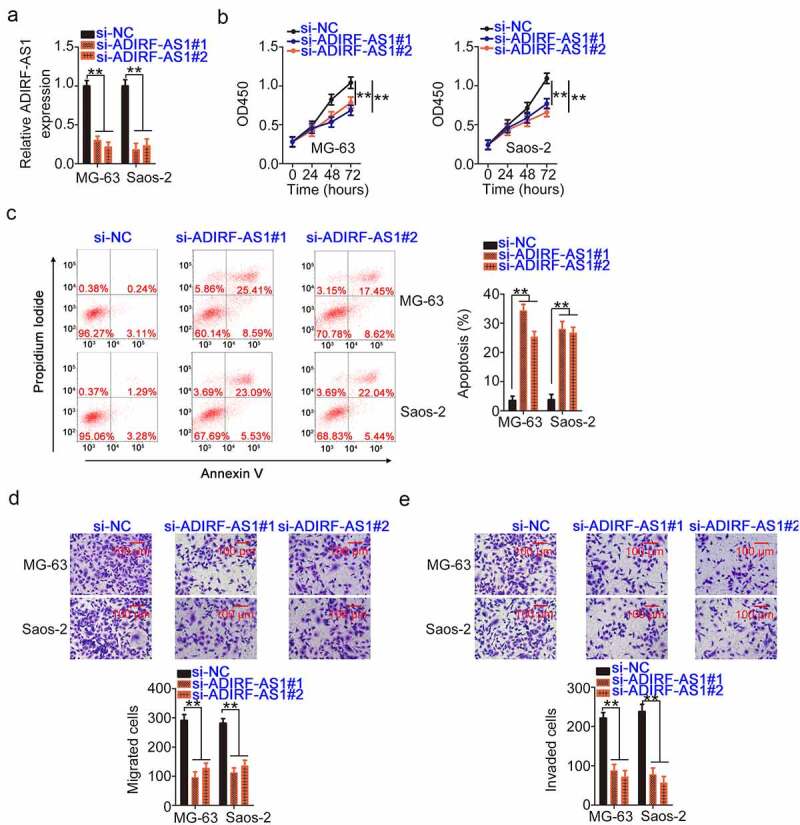
ADIRF-AS1 knockdown restricts the malignancy of OS cells. (a) The interference efficiency of si-ADIRF-AS1#1 and si-ADIRF-AS1#2 in OS cells was validated by qRT-PCR. (b) Effects of ADIRF-AS1 deficiency on OS cell proliferation. (c) The apoptosis of OS cells treated with si-ADIRF-AS1 was examined by flow cytometric analysis. (d, e) The migratory and invasive properties of OS cells transfected with si-ADIRF-AS1 or si-NC. **P < 0.01.

MG-63 and Saos-2 expressed observably higher ADIRF-AS1 levels among the four tested OS cell lines. Therefore, they were used in functional experiments. To determine the roles of ADIRF-AS1 in OS, we induced its depletion in OS cells. To prevent off-target effects, two siRNAs (si-ADIRF-AS1#1 and si-ADIRF-AS1#2) were used, and the interference efficiency was confirmed via qRT-PCR (Figure 2a). OS cell proliferation was evidently hindered in cells transfected with si-ADIRF-AS1 compared with that of those transfected with si-NC (Figure 2b). Additionally, ADIRF-AS1 knockdown promoted the apoptosis of OS cells (Figure 2c). Furthermore, the motility (Figure 2d and e) properties of OS cells were restricted in response to ADIRF-AS1 ablation. Thus, ADIRF-AS1 exerts pro-oncogenic effects in OS cells.

### ADIRF-AS1 serves as a miR-761 sponge

**Figure 3.**
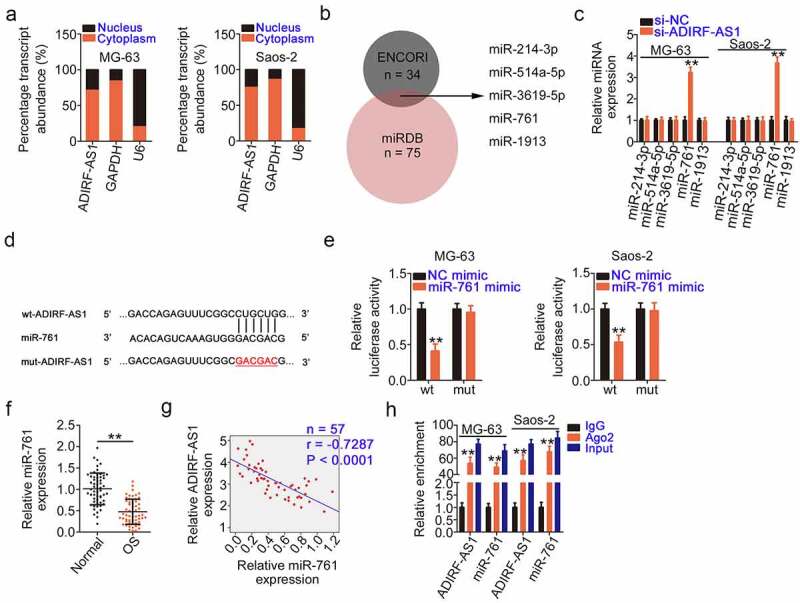
ADIRF-AS1 directly sponges miR-761 in OS. (a) The localization of ADIRF-AS1 in OS cells was demonstrated by subcellular fractionation experiments. (b) The putative targets of ADIRF-AS1 were searched using ENCORI and miRDB. (c) After ADIRF-AS1 knockdown in OS cells, the levels of miR-214–3p, miR-514a-5p, miR-3619–5p, miR-761, and miR-1913 were quantified by qRT-PCR. (d) Schematic representation of the wt and mut binding sequences of miR-761 within ADIRF-AS1. (e) The luciferase activity of wt-ADIRF-AS1 or mut-ADIRF-AS1 was detected after treatment with the miR-761 mimic or NC mimic. (f) The expression levels of miR-761 in OS tissues was determined by qRT-PCR. (g) Pearson’s correlation coefficient analysis was used to test the relationship between ADIRF-AS1 and miR-761 expression in OS tissues. (h) A RIP assay showed that ADIRF-AS1 is present with miR-761 in Ago2-containing immunoprecipitated RNA in OS cells. **P < 0.01.

Cumulative studies have confirmed that lncRNA in the cytoplasm acts as a miRNA sponge or competing endogenous RNAs (ceRNA) [34]. To illustrate the mechanisms responsible for the actions of ADIRF-AS1, the cellular distribution of ADIRF-AS1 in OS cells was studied. As determined by subcellular fractionation, most ADIRF-AS1 was detected in the cytoplasm of OS cells (Figure 3a). A bioinformatics analysis was implemented using the online prediction tools ENCORI and miRDB to identify possible interacting miRNAs that target ADIRF-AS1. Five overlapping miRNAs (Figure 3b) with the potential to interact with ADIRF-AS1 were detected. We measured their expression in ADIRF-AS1-deficient OS cells using qRT-PCR, identifying a significant increase in miR-761 levels, while the levels of the other four miRNAs remained unaltered (Figure 3c).

The wild-type and mutant miR-761 binding sites within ADIRF-AS1 are shown in Figure 3d. As evidenced by a luciferase reporter assay, exogenous miR-761 expression restricted the luciferase activity of wt-ADIRF-AS1 in OS cells did not affect the activity of mut-ADIRF-AS1 (Figure 3e). Additionally, qRT-PCR detected a considerable decrease in miR-761 levels in OS tissues compared with its levels in normal tissues (Figure 3f). Furthermore, ADIRF-AS1 levels in OS tissues negatively correlated with miR-761 levels (Figure 3g). Finally, a RIP assay confirmed that ADIRF-AS1 and miR-761 were enriched in Ago2-containing immunoprecipitated RNA (Figure 3h), which suggests that ADIRF-AS1 and miR-761 are co-expressed in the RNA-induced silencing complex. Altogether, these data show that ADIRF-AS1 can sponge miR-761 in OS.

### IRS1 is controlled by the ADIRF-AS1/miR-761 axis in OS cells

**Figure 4.**
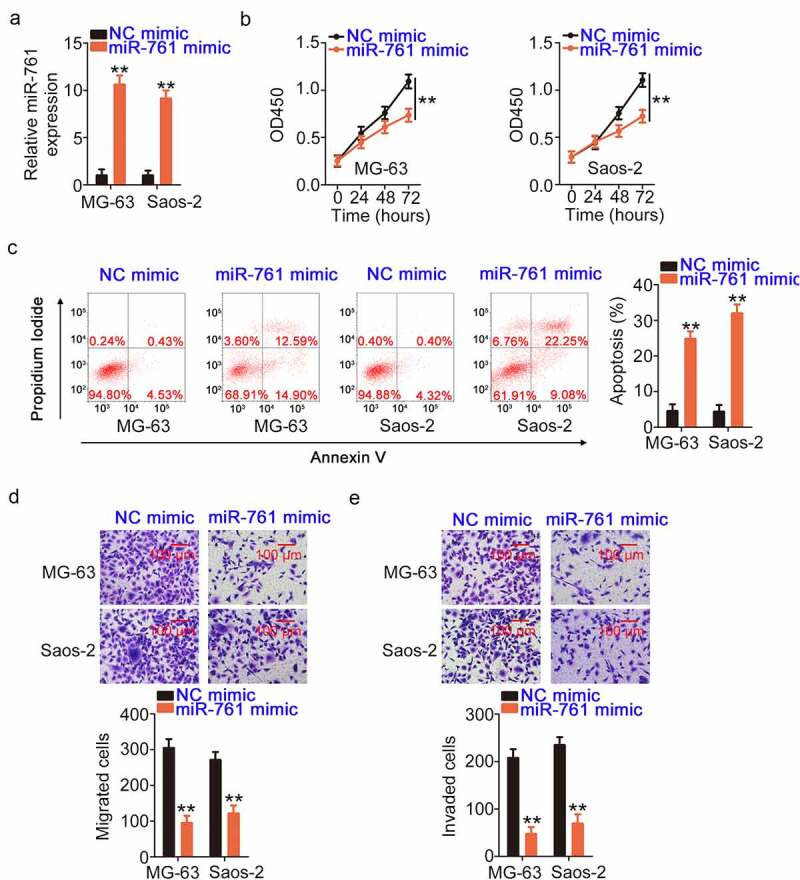
The anti-oncogenic activities of miR-761 in OS cells. (a) Verification of the transfection efficiency of the miR-761 mimic in OS cells. (b, c) The effects of the miR-761 mimic on the proliferation and apoptosis of OS cells. (d, e) The motility of OS cells after overexpression of miR-761. **P < 0.01.

The regulatory actions of miR-761 in OS cells were also investigated. A miR-761 mimic was used to overexpress miR-761 (Figure 4a) and perform functional experiments. miR-761 overexpression strikingly impeded the proliferation of (Figure 4b) and promoted apoptosis (Figure 4c) in OS cells. Migration and invasion (Figure 4d and e) were evidently hindered in OS cells after treatment with the miR-761 mimic.

**Figure 5.**
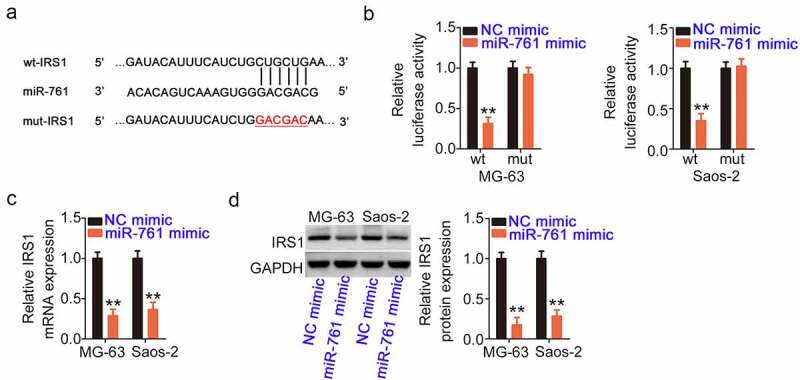
miR-761 directly targets IRS1. (a) The binding sites of miR-761 within the IRS1 3ʹ-UTR. (b) The luciferase activity of wt-IRS1 or mut-IRS1 was measured after treatment with the miR-761/NC mimic. (c, d) The IRS1 levels were assessed in miR-761-overexpressing OS cells. **P < 0.01.

The 3ʹ-UTR of IRS1 harbored a complementary binding site for miR-761 (Figure 5a) and sparked our interest to determine its regulatory roles in OS progression [35–39]. As evidenced by the luciferase reporter assay, the activity triggered by the wt-IRS1 reporter plasmid decreased in miR-761 mimic-transfected OS cells. However, its repressive effect was counteracted when the binding site was mutated (Figure 5b). Moreover, IRS1 mRNA and protein levels were quantified after miR-761 mimic transfection, indicating that miR-761 decreases IRS1 expression in OS cells (Figure 5c and d).

**Figure 6.**
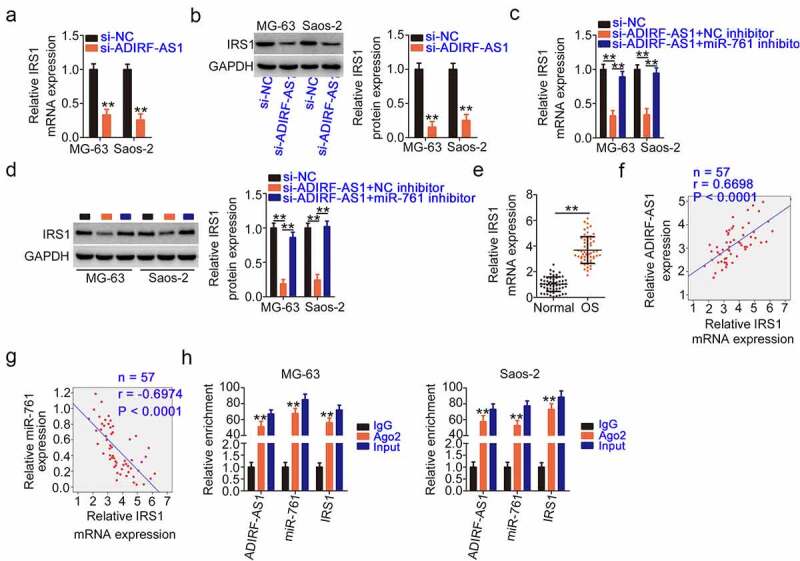
ADIRF-AS1 deficiency decreases IRS1 by siphoning miR-761. (a, b) The expression of IRS1 was determined in OS cells upon ADIRF-AS1 ablation. (c, d) IRS1 expression was detected in ADIRF-AS1-depleted OS cells that were co-transfected with a miR-761 inhibitor. (e) IRS1 expression in OS tissues was measured by qRT-PCR. (f) The relationship between ADIRF-AS1 and IRS1 levels and (g) between miR-761 and IRS1 levels in OS tissues was confirmed by Pearson’s correlation coefficient analysis. (h) The enrichment of ADIRF-AS1, miR-761, and IRS1 in Ago2-containing immunoprecipitated RNA. **P < 0.01.

To determine the regulatory effect of ADIRF-AS1 on IRS1 expression, ADIRF-AS1 was depleted in OS cells, which resulted in a notable decrease in IRS1 expression (Figure 6a and b). Nevertheless, IRS1 reduction due to ADIRF-AS1 silencing could be abrogated by miR-761 inhibition (Figure 6c and d). Furthermore, IRS1 was highly expressed (Figure 6e) and its levels positively correlated with ADIRF-AS1 levels (figure 6f) in OS tissues. Moreover, an inverse relationship was observed between IRS1 and miR-761 expression (Figure 6g). ADIRF-AS1, miR-761, and IRS1 were all enriched in Ago2-containing immunoprecipitated RNA (Figure 6h), which confirmed their presence in the RNA-induced silencing complex. These results suggest that ADIRF-AS1, miR-761, and IRS1 constitute ceRNAs in OS and that ADIRF-AS1 controls IRS1 expression by competitively binding to miR-761.

### miR-761 underexpression or IRS1 overexpression offsets ADIRF-AS1 ablation-induced antitumor activities in OS cells

**Figure 7.**
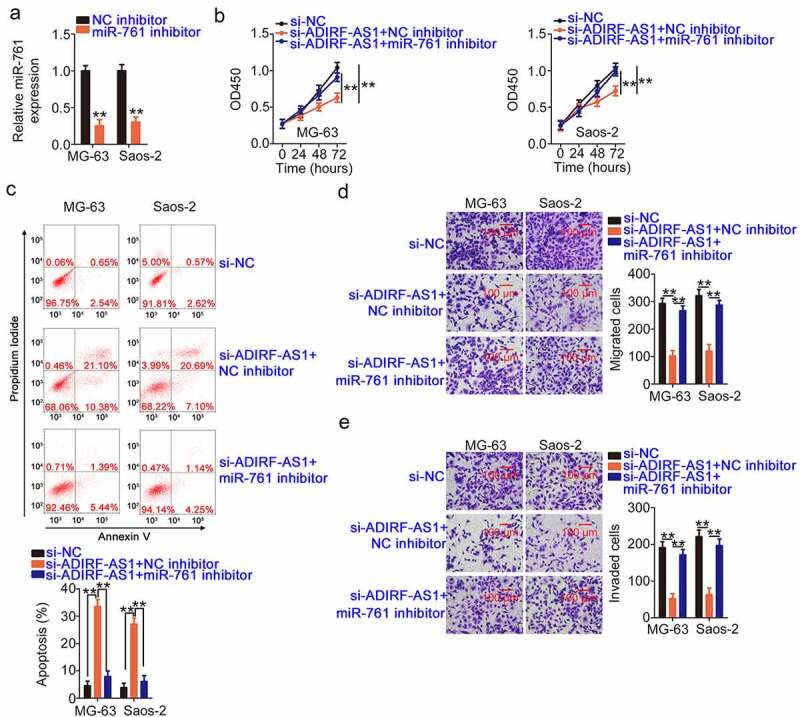
A miR-761 inhibitor counteracts the effects of ADIRF-AS1 underexpression in OS cells. (a) miR-761 levels in OS cells after miR-761 or NC inhibitor treatment were detected by qRT-PCR. (b, c) Cell proliferation and apoptosis were tested in ADIRF-AS1-silenced OS cells after miR-761 inhibitor treatment. (d, e) The migration and invasiveness of the aforementioned cells. **P < 0.01.

After verifying the carcinogenic actions of ADIRF-AS1 and the relationship of ADIRF-AS1 with miR-761 and IRS1, rescue experiments were performed to evaluate functional relationships. A miR-761 inhibitor was used in rescue experiments, and its efficiency in lowering miR-761 levels was validated by qRT-PCR (Figure 7a). After the transfection of ADIRF-AS1-silenced OS cells with a miR-761 inhibitor or a NC inhibitor, cell proliferation and apoptosis were detected by the CCK-8 assay and flow cytometry. The inhibition of proliferation and promotion of apoptosis (Figure 7b and c) of ADIRF-AS1-silenced OS cells were reversed by miR-761 inhibitor treatment. Additionally, the suppression of OS cell migration and invasion induced by si-ADIRF-AS1 transfection was recovered after miR-761 inhibitor cotransfection (Figure 7d and e).

**Figure 8.**
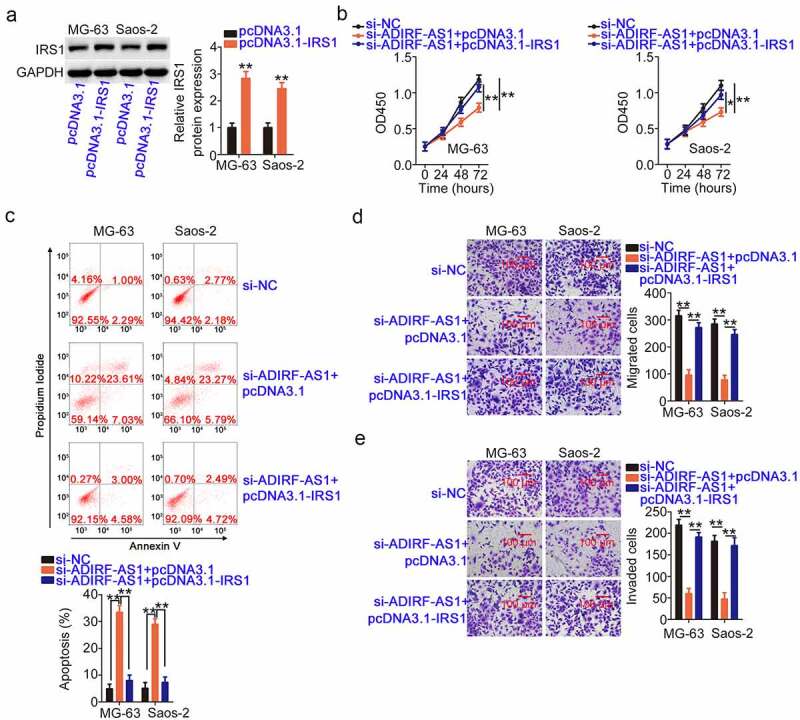
IRS1 overexpression abrogates the inhibitory activity of si-ADIRF-AS1 on OS cells. (a) The efficiency of pcDNA3.1-IRS1 transfection was validated by Western blotting. (b, c) Proliferation and apoptosis were detected in OS cells after si-ADIRF-AS1 and pcDNA3.1-IRS1 or pcDNA3.1 cotransfection. (d, e) The migratory and invasive capacities were examined in treated OS cells as described above. *P < 0.05 and **P < 0.01.

Simultaneously, pcDNA3.1-IRS1 plasmid transfection led to considerable IRS1 overexpression in OS cells (Figure 8a). Increased IRS1 levels abrogated the anti-proliferative (Figure 8b) and pro-apoptotic (Figure 8c) effects of ADIRF-AS1 depletion in OS cells. Furthermore, the migration and invasion (Figure 8d and e) impaired by ADIRF-AS1 deficiency were recovered following IRS1 re-expression. Collectively, these data indicate that the ADIRF-AS1/miR-761/IRS1 pathway regulates the cellular performance of OS cells.

### Depleted ADIRF-AS1 restricts tumor growth in vivo

**Figure 9.**
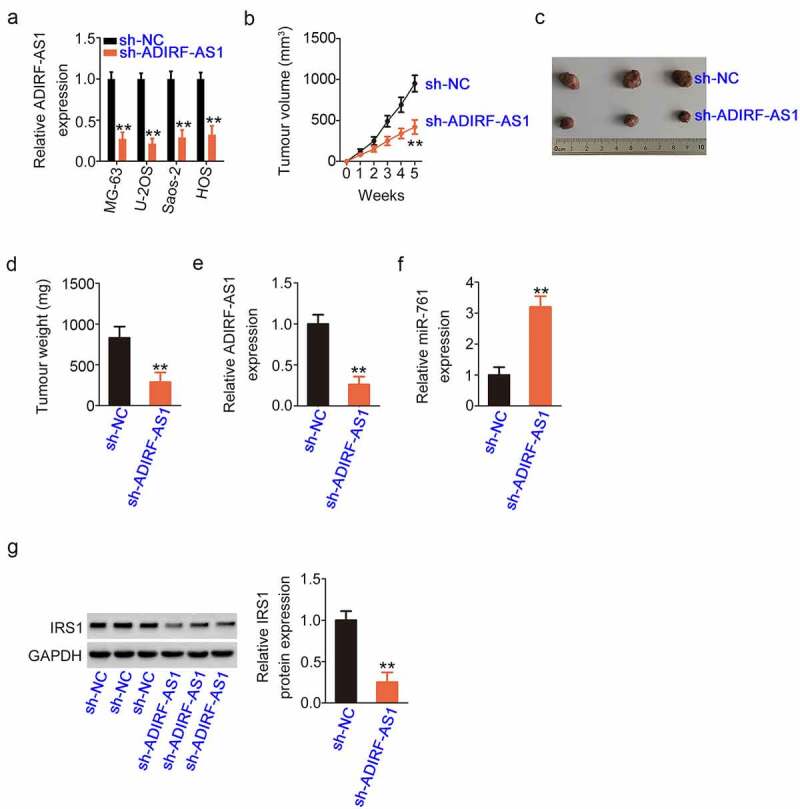
Silencing ADIRF-AS1 impedes cell growth *in vivo*. (a) The knockdown efficiency of sh-ADIRF-AS1 in the four OS cell lines. (b) The growth of ADIRF-AS1-deficient xenografts was lower than that of the control group. (c) Pictures of xenografts collected from both groups. (d) The weight of xenografts in the sh-ADIRF-AS1 group was lower than that of xenografts in the control group. (e, f) qRT-PCR demonstrated a notable decrease in ADIRF-AS1 and an increase in miR-761 in xenografts with stable ADIRF-AS1 ablation. (g) Western blotting revealed the obvious downregulation of IRS1 protein in ADIRF-AS1-depleted xenografts. **P < 0.01.

The anti-growth effect of ADIRF-AS1 knockdown on OS cells was demonstrated *in vivo* using xenograft experiments. Prior to that, we determined the knockdown efficiency of sh-ADIRF-AS1, which was the highest in. U-2OS cells. Accordingly, U-2OS cells were chosen for xenograft experiments (Figure 9a). The growth of sh-ADIRF-AS1-transfected xenografts was notably suppressed in comparison with that in sh-NC-transfected xenografts (Figure 9b). The xenografts excised from sh-ADIRF-AS1-injected mice were strikingly smaller (Figure 9c) and lighter (Figure 9d) than those from sh-NC-injected mice. Moreover, there was downregulated ADIRF-AS1 (Figure 9e) and increased miR-761 (figure 9f), as detected by qRT-PCR, and decreased IRS1 protein (Figure 9g) in xenografts obtained from sh-ADIRF-AS1-injected mice compared with those from sh-NC-injected mice. Therefore, ADIRF-AS1 knockdown weakens the growth capacity of OS cells *in vivo*.

## Discussion

There is vast evidence that lncRNAs exert critical regulatory roles in OS [40–42]. The modulation of key signaling pathway genes by lncRNAs is also implicated in OS occurrence and progression [22]. However, the contribution of abundant lncRNAs to OS pathogenesis had not been clarified until now and requires further exploration. Herein, our findings validated that ADIRF-AS1 plays carcinogenic roles in OS by affecting miR-761/IRS1, providing evidence for the development of novel modalities of drug administration in anticancer therapeutics.

LncRNAs have attracted considerable attention in recent years. For instance, the lncRNAs UCA1 [43], FGD5-AS1 [44], and NEAT1 [45] are upregulated in OS and exacerbate osteosarcomagenesis. On the contrary, H19 [46], TUSC7 [47], and LINC00691 [48]are lowly expressed in OS and inhibit oncogenicity. Nevertheless, the detailed functions of ADIRF-AS1 in OS have not been fully elucidated. In this study, ADIRF-AS1 was confirmed to be overexpressed in OS. Specifically, the overall survival of OS patients with high ADIRF-AS1 levels was shorter than that of those with low ADIRF-AS1 levels. ADIRF-AS1 knockdown led to restricted proliferation, migration, and invasiveness and increased apoptosis in OS cells. Additionally, ADIRF-AS1 downregulation impeded tumor growth *in vivo*. Our results may help to provide an effective reference for the clinical management of OS.

Mechanistically, lncRNAs participate in physiological and pathological processes in different ways largely determined by their localization [49]. Regarding cytoplasmic lncRNAs, the ceRNA theory has been extensively researched, having shown that lncRNAs contain miRNA response elements and competitively bind to certain miRNAs, ultimately decreasing the repression of downstream genes by miRNAs [34]. The role of lncRNAs in modulating the aggressiveness of OS cells is associated with the complex crosstalk among multiple RNAs in the ceRNA network [50,51]. The lncRNA/miRNA/mRNA pathway, which is regulated by ceRNA, supplements the roles of miRNAs [52].

The ceRNA regulatory pathway provides a new perspective that can clarify the molecular events of ADIRF-AS1 engaged in OS. Initially, we determined the cellular location of ADIRF-AS1 in OS cells. Using subcellular fractionation, our data showed that ADIRF-AS1 was abundant in both the nucleus and cytoplasm and that the cytoplasm contained more ADIRF-AS1. Considering that lncRNAs may function as ceRNAs or molecular sponges, a bioinformatics analysis was performed to predict ADIRF-AS1-miRNA. We identified a miR-761-binding site in the ADIRF-AS1 sequences and employed a luciferase reporter assay and RIP to confirm the target binding effect. Subsequently, mechanistic studies successfully demonstrated that IRS1 is a direct target of miR-761 in OS. Next, ADIRF-AS1 was confirmed to exert positive regulatory activity on IRS1, since when ADIRF-AS1 was knocked down in OS, miR-761 was upregulated and IRS1 was downregulated. Furthermore, ADIRF-AS1, miR-761 and IRS1 were all significantly expressed in OS tissues. Altogether, the three RNAs, ADIRF-AS1, miR-761 and IRS1, constitute a novel ceRNA regulatory axis in OS. miR-761 is upregulated in hepatocellular carcinoma [53], breast cancer [54], and gastric cancer [55] and exerts cancer-suppressing effects. In contrast, miR-761 is downregulated in ovarian cancer [56], colorectal cancer [57,58], and OS [59–61] and plays a carcinogenic role. These observations imply that the expression profile and functions of miR-761 display tissue specificity in human cancers. In agreement with previous studies [59–61], our research also authenticated miR-761 as an anti-oncogenic miRNA in OS.

Furthermore, IRS1, as a mediator of oncogenic insulin-like growth factor signaling, was verified to be a direct downstream target of miR-761. During osteosarcomagenesis and progression, IRS1 executes important regulatory functions and participates in the control of various tumor-associated malignant activities [35–39]. Using a rescue experiment, we found that miR-761 underexpression or IRS1 restoration could neutralize the effects of ADIRF-AS1 ablation in OS. Accordingly, the ADIRF-AS1/miR-761/IRS1 pathway was acknowledged as a promoter of OS malignancy, and miR-761/IRS1 was characterized as the downstream effector of ADIRF-AS1.

In the last decade, multiple inhibitors targeting lncRNA have been revealed to promote cancer regression [62–64]. However, only a very small fraction have shown clinical relevance. In recent years, the use of antisense oligonucleotides (ASOs) has provided novel insight into cancer diagnosis and treatment [65]. For instance, a lncRNA called prostate cancer antigen 3 (PCA3) has been applied in clinical practice as a biomarker for prostate cancer diagnosis [66]. Therefore, lncRNAs have considerable potential as diagnostic biomarkers and therapeutic targets in OS for its early diagnosis and management.

Herein, we used two OS cell lines, MG-63 and Saos-2, to explore the regulatory activities and underlying mechanisms of ADIRF-AS1 in OS. However, the 143B cell line, which presented high aggressiveness and the ability to metastasize, was not adopted for performing functional experiments, consisting a. limitation of our study that, will be resolvedresovle it in the near future.

## Conclusion

Briefly, we demonstrated that ADIRF-AS1 exacerbates the oncogenicity of OS cells by targeting the miR-761/IRS1 axis, in which ADIRF-AS1 acts as a ceRNA for miR-761 and consequently leads to IRS1 overexpression. Our findings may aid the development of lncRNA-directed therapeutics for OS.

## Highlights

ADIRF-AS1 was overexpressed in OS and was closely related to patients’ overall survival.

ADIRF-AS1 exacerbated the oncogenicity of OS cells *in vitro* and *in vivo*.

miR-761/IRS1 acted as the downstream effector of ADIRF-AS1.
